# Anaplastic thyroid carcinoma with unusual long-term survival: a case report

**DOI:** 10.1186/s13256-021-03249-8

**Published:** 2022-02-01

**Authors:** Fany Moreno, Clarisa Reyes, César Alas Pineda, Gustavo Castellanos, Flory Cálix, Jorge Calderón, Walter O. Vasquez-Bonilla

**Affiliations:** 1 Departamento de Oncología, Hospital Dr. Mario Catarino Rivas, San Pedro Sula, Cortés Honduras; 2 Hospital Bendaña, San Pedro Sula, Cortés Honduras; 3 ASOCEM Universidad Católica de Honduras – San Pedro y San Pablo (ASOCEM UNICAH-SPSP), San Pedro Sula, Cortés Honduras; 4 Facultad de Medicina y Cirugía, Universidad Católica de Honduras – Campus San Pedro y San Pablo, San Pedro Sula, Cortés Honduras; 5 Departamento de epidemiología, Hospital Dr. Mario Catarino Rivas, San Pedro Sula, Cortés Honduras; 6 Facultad de Medicina y Cirugía, Universidad Autónoma de Honduras en el Valle de Sula, San Pedro Sula, Cortés Honduras; 7 Departamento de Patología, Hospital General San Juan de Dios, Guatemala, Guatemala

**Keywords:** Anaplastic carcinoma, Thyroidectomy, Thyroid neoplasms, Case report

## Abstract

**Background:**

Anaplastic thyroid carcinoma is a rare, rapidly progressive, and highly aggressive tumor. It has a global annual incidence of 1–2 per million people. It mostly affects older adults and women. The median survival duration after diagnosis does not exceed 6–8 months.

**Case presentation:**

A 60-year-old female patient of mixed race (Honduran) presented to the local medical service with dysphonia that had started approximately 2 months earlier, accompanied by orthopnea that had started 1 month earlier. On physical examination, a soft mass was palpated within the anterior neck region; it was approximately 4 cm in diameter, painless, and mobile on swallowing, and had irregular margins. Ultrasound and computed tomography of the neck were performed. Subsequently, fine needle aspiration biopsy was performed. The histological diagnosis was anaplastic thyroid carcinoma (stage IVB). She underwent total thyroidectomy and chemotherapy. She is currently in her fifth year of remission after diagnosis and remains under oncologic surveillance.

**Discussion:**

Anaplastic thyroid carcinoma demonstrates a lethal behavior. Approximately 18% survive for more than a year after diagnosis, and 0–10% survive for 5 years. Different pretherapeutic prognostic factors may affect survival, including age < 70 years, the absence of distant metastases, and complete local resection.

**Conclusion:**

Conventional treatment improves the quality of life of the patient, but the results are not encouraging for the medium and long term. Only a few patients manage to exceed the average life expectancy of 3–6 months, despite undergoing the currently available therapeutic regimen.

## Background

Anaplastic thyroid carcinoma (ATC) is a rare undifferentiated neoplasm characterized by a sudden onset and rapid progression. It has a global annual incidence of 1–2 per million people and accounts for approximately 1–2% of thyroid neoplasms [1], with rates of disease-specific mortality that go up to 100% [4].

ATC is associated with genetic alterations that cause the dysregulation of the MAPK and PI3K/AKT signaling pathways, which affects the *BRAF* and *KRAS* and *BRAF* and *PTEN* genes, among others, known for important modulatory functions of cell growth, survival, and proliferation. The risk factors associated with the development of this oncological process are unknown, and its etiology is still uncertain [2].

Undifferentiated thyroid cancer usually arises from well-differentiated thyroid tumors; with the ongoing process of dedifferentiation, the thyroid tumors lose the characteristics of the cells from which they originated, and this reaches the point where essential features, such as iodine uptake and thyroglobulin production, are absent or diminished [2, 3].

ATC mostly affects individuals between the sixth and seventh decade of life, with a greater predisposition for the female sex. It is extremely aggressive, and its prognosis is unfavorable; it accounts for approximately half of the deaths caused by all thyroid cancer processes. The average survival duration of patients with ATC is 3–6 months; approximately only 18% of cases may survive for more than a year after diagnosis [4].

Total thyroidectomy, chemotherapy, and radiation therapy are used to manage ATC; however, no significant reduction in mortality caused by tumorigenesis has been reported despite the implementation of appropriate measures. The choice of treatment depends on the characteristics of the tumor and the patient [5].

The relevance of the case described below lies not only in the rarity of anaplastic thyroid carcinoma, but also in the favorable evolution with an unusually long survival in a patient in the sixth decade of life, treated in a public hospital in Honduras, where, despite the economic and technical limitations of the public health system, the patient considerably improved her quality of life, having no recurrence of the disease.

## Case presentation

We present the case of a 60-year-old female patient of mixed race (Honduran) from an urban area of Honduras, with a pathological history of arterial hypertension and type 2 diabetes mellitus under control (metformin 500 mg and enalapril 10 mg). In December 2015, she presented to a local medical service with dysphonia that had progressed over 2 months; it was exacerbated by singing and accompanied by orthopnea that had progressed over a month. She denied odynophagia, weight loss, dysphagia, and hyporexia. She had no relevant family history.

Physical examination showed a good general condition, mesomorphic biotype, and stable vital signs. During the segmental physical examination, a soft mass was palpated within the anterior region of the neck; it was painless and mobile on swallowing and had irregular margins without inflammatory signs.

Ultrasound (US) of the neck showed a solid ovoid tumor located in the left thyroid lobe. It had well-defined borders, an approximate dimension of 37 × 24 × 37 mm, and a volume of 17 mL. The right thyroid lobe was normal with a dimension of 34 × 11 × 10 mm and a volume of 2 mL. The thyroid isthmus and adjacent vascular structures had no obvious abnormalities. One week later, fine needle aspiration (FNA) biopsy was performed on a single nodule. FNA was also performed for some lymph nodes suggestive of malignancy. The anatomopathological report confirmed ATC. Laboratory tests showed elevated thyroid stimulating hormone levels (Table 1).

**Table 1:** Laboratory tests 1

Parameter	Values	Reference values
Triiodothyronine (T3)	1.10 ng/mL	0.58–1.59 ng/mL
Thyroxine (T4)	5.47 ug/dL	4.87–11.72 ug/dL
Thyroid stimulating hormone (TSH)	6.69 ulU/mL	0.35–4.94 ulU/mL
Glucose	180 mg/dL	70–110 mg/dL

Computed tomography of the neck performed a month later showed that the thyroid mass covered the entire left lobe, with predominant vascularity toward the periphery. The tumor extended to the isthmus and the right lobe, where the neoplasm replaced the inferior pole. There was also evidence of multiple adenopathies with loss of morphology, and all were larger than 8 mm. There were findings suggestive of infiltration of the perithyroid muscles and displacements of the trachea and carotid sheath; there was no infiltration of the other adjacent structures.

**Fig. 1:**
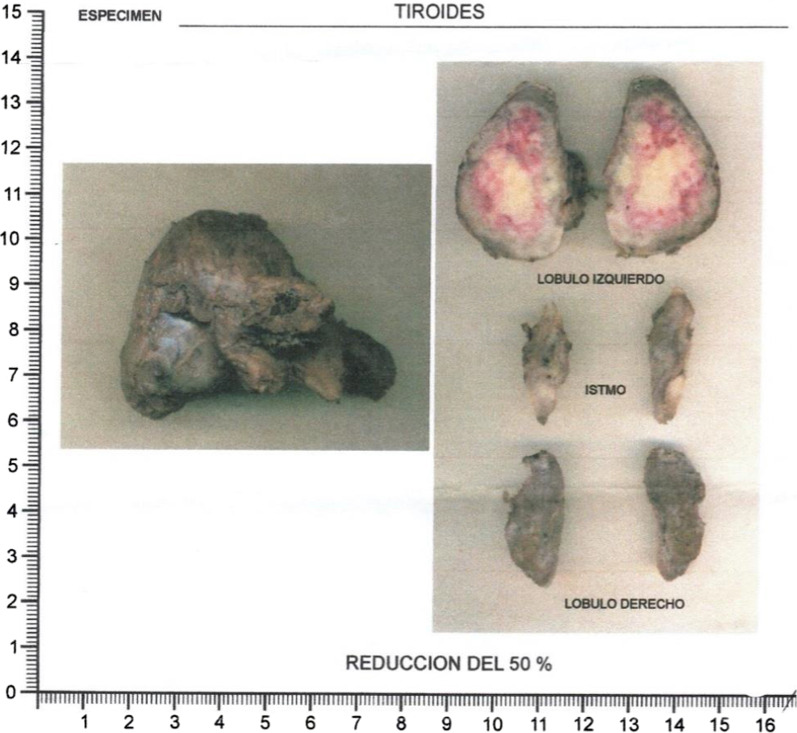
Surgical specimen: 55 × 30 × 20 mm left thyroid lobe, with irregular fibrous aspects on the anterior surface and a ruptured capsule, soft consistency and a mass replacing the entire lobe, without viable thyroid tissue. Right thyroid lobe 25 × 10 mm, with small areas of light brown color on dissection

**Fig. 2:**
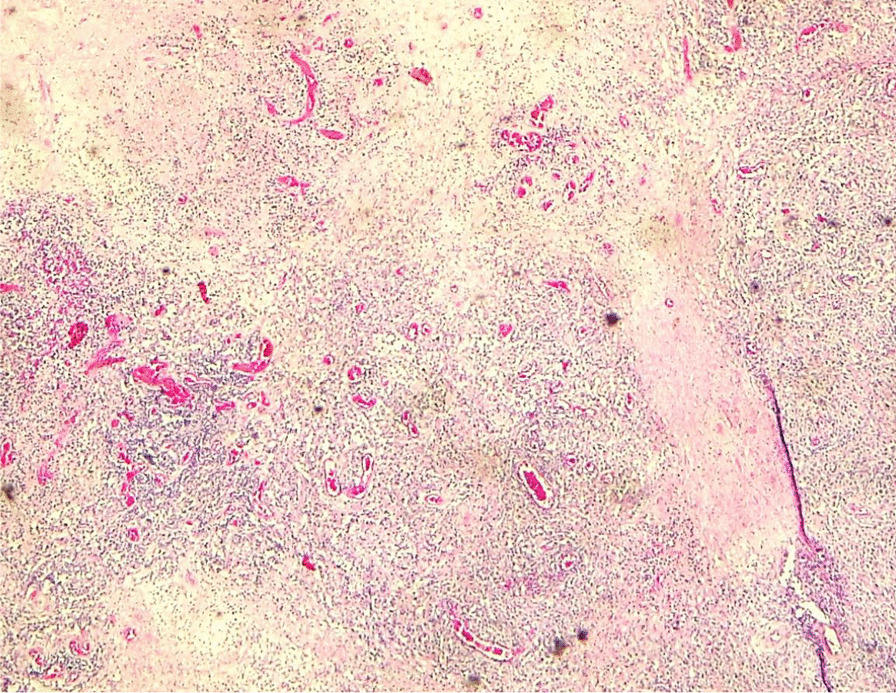
At low magnification, bands of sclerosis are observed interspersed with areas with little neoplastic cells (4×)

**Fig. 3:**
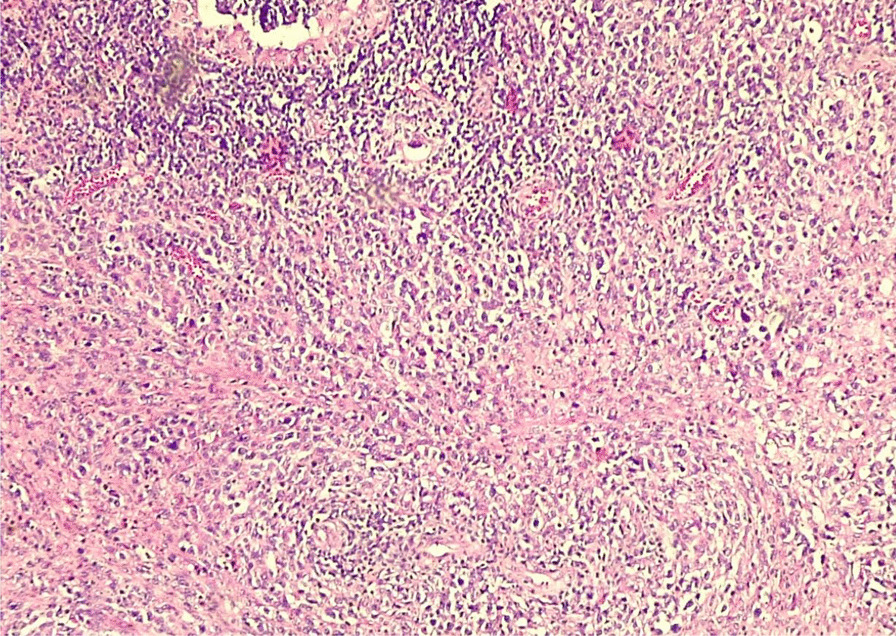
Neoplastic cells of medium to large size, with nuclear pleomorphism and little cytoplasm, immersed in a fibrous stroma and intermixed inflammatory cells composed mainly of lymphocytes arranged in patches (10×)

**Fig. 4:**
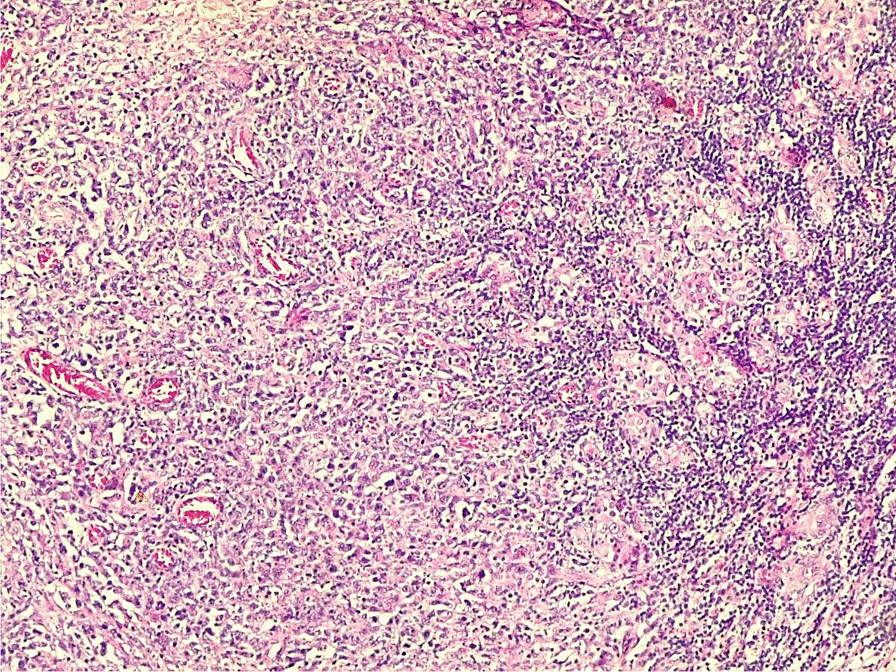
Shows a tumor area on the left and a lymphocytic reaction on the right. There are abundant single tumor cells, some with centroblastic appearances and macronucleoli. There are occasional tubes and acini (40×)

**Fig. 5:**
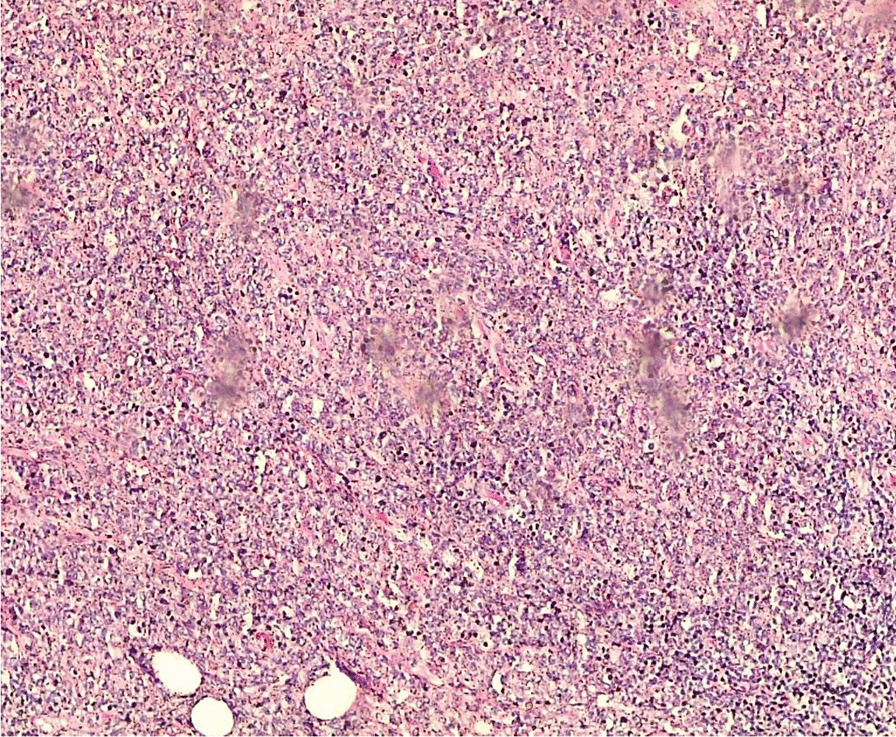
Shows a background of cellular detritus, in addition to a high mitotic rate and neutrophilic activity in the background (40×)

A total thyroidectomy and bilateral central radical dissection were performed in February 2016 in a second-level care unit and sent for a pathological study. The macroscopic description of the right thyroid lobe measuring 25 × 10 mm is as follows: the external surface is rough partially covered by fibrous adhesions, at the cut of a soft consistency of gray color, with small areas of light brown color, separated by the isthmus where colored areas are observed light brown and left thyroid lobe measuring 55 × 30 × 20 mm, external surface is rough covered by fibrous adhesions, when cut with a mass that replaces the entire lobe, without viable thyroid tissue (see Fig. 1). Microscopic study revealed an anaplastic thyroid carcinoma (stage IV B) with focal involvement of soft tissues and lymph nodes with macrometastasis (see Figs. 2, 3, 4, and 5). There were no postoperative complications. No immunohistochemical study was performed.

Subsequently, five cycles of chemotherapy with doxorubicin (60 mg/m^2^) were completed in 6 months. She did not receive any other form of therapy. Currently, the patient is under oncological surveillance (control appointments with imaging studies and hormonal tests every 6 months since her intervention). The most recent follow-up appointment was in February 2021; she showed no signs of recurrence. She has achieved 5 years of complete remission and has maintained optimal health.

## Discussion

ATC is one of the most aggressive cancers, and is responsible for up to half of all deaths caused by thyroid cancers. It accounts for approximately 33–50% of all thyroid neoplasm-related deaths [6].

Five to ten percent of patients survive for approximately 5 years, and the median life expectancy after diagnosis does not exceed 3–6 months [7, 8]. Our patient has exceeded the life expectancy for ATC, having been in complete remission for 5 years after treatment, and this represents a surgical and therapeutic success for the atypical histopathological manifestation.

ATC presents as a solid central mass in the anterior cervical region. Only 10% of patients have the tumor confined to the thyroid gland; 44% and 40% of patients have additional thyroid extension and lymph node metastases, respectively, and the remainder have distant metastases [6]. Clinically, it is accompanied by dysphonia, dysphagia, dyspnea, and cough. Vocal cord paralysis occurs in only 30%, and regional lymph node metastasis occurs in 40% of patients [9].

According to Chintakuntlawar *et al*. [10], 20–50% of ATCs originate from preexisting well-differentiated thyroid carcinomas. Our patient had no history of previous thyroid carcinoma or associated thyroid complications; this was not usual based on the reports of other series for this atypical histological presentation. Immunohistochemistry can be used for a precise diagnosis, but because of the lack of resources, our diagnosis was made purely on clinical and pathological findings.

In a multicenter retrospective study conducted in Germany, it was concluded that the pretherapeutic prognostic factors underlying long-term survival were age < 70 years, the absence of distant metastases, and complete local resection [11].

Our patient had these three prognostic factors: she was 60 years old, did not have distant metastases, and even though she had local invasion of perithyroid soft tissues and lymph nodes, complete resection of the tumor was successfully performed.

ATC is always classified as stage IV. It can be subclassified based on the degree of invasion of surrounding tissues, that is, IVa, IVb, IVc, and IVd [12]. The tumor in our patient adhered to the perithyroid muscles and regional lymph nodes, and was graded as stage IVb.

Wendler *et al*. [11] conducted a multicenter study of 100 patients diagnosed with ATC between 2000 and 2015 at five German referral centers; they demonstrated that survival associated with ATC, independent of treatment, is related to the stage. Their reported survival duration results were as follows: 26 months and 66% in stage IVA, 11 months and 39% for stage IVB, and 3 months and 13% in stage IVC [11]. The patient was diagnosed when the tumor had progressed to stage IVB. In contrast to the report by a German study, our patient has survived for more than 5 years.

Conventional treatment is multimodal, and includes surgical resection, hyperfractionated accelerated external beam radiotherapy, chemotherapy sessions, and palliative care [13]. The intervention is aimed at slowing down the rapid progression of the disease and facilitating better quality of life of the patient.

Thyroidectomy with wide margins is recommended when the tumor is confined to the thyroid parenchyma and it has favorable prognostic features; unilobar, diameter < 5 cm, without spread so that lobectomy can be performed. The intended goal of the procedure is R0 resection with a negative margin [14].

The tumor in our patient extended to the entire left lobe, right lobe, and isthmus; despite this, a total thyroidectomy and bilateral radical central dissection were performed, leaving negative margins. Only chemotherapy was included in the treatment, because the hospital in which the patient was treated did not have access to radiotherapy and the patient could not afford a private unit.

The guidelines of the American Thyroid Association (ATA) recommend surgical resection for patients with stage IVa–IVb disease. Consistent with the literature, the stage of anaplastic carcinoma in this case presentation was IVb. The tumor was successfully resected surgically, and our patient had favorable survival, no recurrence, and a significantly improved quality of life [13].

## Conclusion

Anaplastic carcinoma is the rarest and an extremely aggressive thyroid cancer, accounting for approximately half of all deaths caused by neoplasms of the thyroid gland, with a median 1-year survival rate of only 10–20% according to the World Health Organization (WHO). Several mutations underlie the malignancy of ATC, and therapies targeted at these are being developed. However, while current treatment facilitates improvement in the quality of life of patients, the results are not encouraging in the long term. Only a few patients manage to surpass the average life expectancy.

## Data Availability

The datasets used and/or analyzed during the current study are available from the corresponding author on reasonable request.

## Appendix

See Figs. 1, 2, 3, 4, 5.

## Abbreviations

ATC: Anaplastic thyroid carcinoma
USG: Ultrasound
FNA: Fine needle aspiration biopsy
TSH: Thyroid stimulating hormone
UICC: Union for International Cancer Control
